# Investigating factors associated with the number of rehospitalizations among patients with schizophrenia disorder using penalized count regression models

**DOI:** 10.1186/s12874-022-01648-z

**Published:** 2022-06-15

**Authors:** Mahya Arayeshgari, Ghodratollah Roshanaei, Ali Ghaleiha, Jalal Poorolajal, Leili Tapak

**Affiliations:** 1 Department of Biostatistics, School of Public Health, Hamadan University of Medical Sciences, Hamadan, Iran; 2 Modeling of Noncommunicable Diseases Research Center, Hamadan University of Medical Sciences, Hamadan, Iran; 3 Department of Psychiatry, School of Medicine, Hamadan University of Medical Sciences, Hamadan, Iran; 4 Research Center for Behavioral Disorders and Substance Abuse, Hamadan University of Medical Sciences, Hamadan, Iran; 5 Department of Epidemiology, School of Public Health, Hamadan University of Medical Sciences, Hamadan, Iran

**Keywords:** Count Regression Model, Variable Selection, Minimum Concave Penalty, Random Forest, Rehospitalization, Schizophrenia

## Abstract

**Background:**

Schizophrenia is a chronic, severe, and debilitating mental disorder always considered one of the recurrent psychiatric diseases. This study aimed to use penalized count regression models to determine factors associated with the number of rehospitalizations of schizophrenia disorder.

**Methods:**

This retrospective cohort study was performed on 413 schizophrenic patients who had been referred to the Sina (Farshchian) Educational and Medical Center in Hamadan, Iran, between March 2011 and March 2019. The penalized count regression models were fitted using R.3.5.2.

**Results:**

About 73% of the patients were male. The mean (SD) of age and the number of rehospitalizations were 36.16 (11.18) years and 1.21 (2.18), respectively. According to the results, longer duration of illness (*P* < 0.001), having a positive family history of psychiatric illness (*P* = 0.017), having at least three children (*P* = 0.013), unemployment, disability, and retirement (*P* = 0.025), residence in other Hamadan province townships (*P* = 0.003) and having a history of arrest/prison (*P* = 0.022) were significantly associated with an increase in the number of rehospitalizations.

**Conclusion:**

To reduce the number of rehospitalizations among schizophrenic patients, it is recommended to provide special medical services for patients who do not have access to specialized medical centers and to create the necessary infrastructure for the employment of patients.

**Supplementary Information:**

The online version contains supplementary material available at 10.1186/s12874-022-01648-z.

## Background

Schizophrenia is a kind of mental disorder characterized by distortions in thought, speech, perception, emotion, sense of self, and behavior. In addition, hallucinations and delusions are the general symptoms of this disease [1]. This illness is the 11th leading cause of disability worldwide [2]. This chronic and severe mental disorder has currently affected about 20 million people worldwide and its annual incidence has been estimated at 1 million [3]. About 90% of all people with untreated schizophrenia live in low and middle-income countries [1]. The prevalence of psychotic disorders (such as schizophrenia) has been reported at 0.89% in Iran [4]. However, according to the World Health report, 17% of the psychiatric hospitalizations in Iranian hospitals are for people with schizophrenia [5]. The prevalence of schizophrenia is the same for men and women, however, its occurrence is earlier for men and generally has a better outcome for females compared to males. Schizophrenia has been reported to be more common in people born during the winter and early spring, which may be due to factors such as the virus or changes in eating habits. Social pressures in urban areas also increase the risk of suffering from schizophrenia for individuals who are prone to this disease [6]. Premature mortality in people with schizophrenia is 2 to 3 times higher than in the general population [7]. It often occurs due to preventable physical ailments (such as cardiovascular disease) [1]. Suicide and nicotine dependence are other causes of death among these patients.

Schizophrenia is a type of recurrent disease, and even if antipsychotics are prescribed, the chance of readmission is about 40 to 60 percent within two years after the first admission. More than 50% of these patients are frequently hospitalized, have worsening symptoms, experience major mood disorders, and have several attempts for suicide [6]. Identifying the factors associated with the number of rehospitalizations provides valuable results for healthcare planning and rehospitalization prevention.

Statistical count regression models including Poisson regression (PR) and Negative Binomial (NB) as well as zero-inflated Poisson (ZIP), Hurdle, and zero-inflated Negative Binomial (ZINB) models in the presence of over-dispersion or extra zeros can be used to determine the correlates of the number of rehospitalizations. However, one main issue in statistical modeling is the variable selection and achieving an interpretable model. There are several methods for selecting a variable, including classical methods, such as stepwise regression and the best subset selection. These methods generally require very complex and time-consuming calculations, but their most considerable drawback is instability [8]. It means by making the slightest change in the data, the selected model may be different, which in turn reduces the accuracy of the predictions. To solve these problems, a penalty term can be added to the likelihood function which performs variable selection and coefficients estimation simultaneously [9]. The penalized version of count regression models using popular penalties including Adaptive Least Absolute Shrinkage and Selection Operator (ALASSO) penalty [10], Smoothly Clipped Absolute Deviation (SCAD) penalty [11], and Minimum Concave Penalty (MCP) [12] has been developed well. It has been proved that all these penalties select true variables associated with outcome.

This study was conducted to identify factors associated with the number of rehospitalizations of schizophrenia disorder using penalized count regression models with ALASSO, SCAD, and MCP penalties. We also considered Backward Stepwise and Random Forest (RF) methods and compared their performances through a simulation study. We finally fitted a regression model on a real dataset of schizophrenic patients with variables selected by a more performing method found by simulation.

## Methods

### Data

In this retrospective cohort study (approved by “The Ethical Committee of the Hamadan University of Medical Sciences”; NO. IR.UMSHA.REC.1398.075), all records of patients with schizophrenia hospitalized at Sina Hospital (Farshchian) in Hamadan, Iran, from March 2011 to March 2019 were reviewed, and finally, 413 patients were selected according to inclusion criteria. All methods were carried out by relevant guidelines and regulations. Inclusion criteria were: the diagnostic criteria for schizophrenia in ICD-10 (International Classification of Diseases, version 10), patients without change of disease in each hospitalization, and patients without other psychiatric disorders at the same time. To extract the information from the patients’ files (without patient involvement), a checklist made by the researchers and a clinical consultant was used. In this checklist, the independent variables were divided into two parts: demographic and clinical characteristics, which were:

- Demographic characteristics: age, gender, birth season, the birthplace township, education status, marital status, number of children, employment status, residence status, the township of residence, living status, having homogeneous siblings, number of siblings, history of emotional distress, including illness or death of a family member or loved one, parental divorce, marriage, pregnancy, emotional breakdown, etc., history of arrest or prison, history of substance abuse, including opium, heroin, cannabis, methamphetamine, crack, and so on (based on a compilation of interviews with patients and their companions and laboratory results), and a history of smoking. In addition, to determine the population of the city or village where people live, the results of the general population and housing censuses in 2011 and 2016 were used.
- Clinical characteristics: age at onset of illness, duration of illness, family history of psychiatric illness, history of medical disease, including cardiovascular, visual and auditory diseases, skin, hypertension, hyperlipidemia, glands, gastrointestinal, neurological, rheumatological and urological, history of non-adherence to antipsychotic drugs (including failure to take medication, taking lower doses than prescribed, and premature termination of medication), and history of the suicide attempt.

The variable of the number of psychiatric rehospitalizations of schizophrenic patients (from the onset of the disease to the end of the study (March 2019)) was considered as the response variable. It is noteworthy that variables were measured at the time of the last recurrence of the symptoms of the patients’ disease. Descriptive statistics of the response variable and characteristics of the studied schizophrenic patients are presented in Tables 2, 3 and 4.

### Statistical analysis

Variable selection methods including ALASSO, SCAD, MCP, Backward Stepwise, and RF were used through a simulation study by generating Negative Binomial distribution for the response variable. Methods were compared and the best one was used to fit on schizophrenia data to determine variables related to the number of psychiatric rehospitalization for schizophrenia disorder. The best model also was compared to other models such as zero-inflated and full models using schizophrenia data.

### Poisson regression (PR) model

Poisson regression is the first model used specifically for modeling count data and also is considered as the basis for many count models [13]. If $Y_{i}$ ($i = 1,...,n$) is a discrete random variable and follows the Poisson distribution with the parameter $\mu_{i}$ and $n$ is the sample size, then its density function is defined as follows:

1 $$f(Y_{i} = y_{i} ;\mu_{i} ) = \frac{{(e^{{ - \mu_{i} }} )\,(\mu_{i}^{{y_{i} }} )}}{{y_{i} !}}\,\,\,\,\,\,;y_{i} = 0,1,2,...\,$$

With equal mean and variance: $E(Y_{i} ) = V(Y_{i} ) = \mu_{i}$.

In the Poisson regression model, a logarithmic link function is typically used to link $\mu_{i}$ to the linear predictor $X_{i}$:

2 $$\log (\mu_{i} ) = X_{i}^{T} \beta$$

where the $X_{i} = (1,X_{i1} ,X_{i2} ,...,X_{ip} )^{T}$ shows the vector of predictor variables for the ith subject, $\beta = (\beta_{0} ,\beta_{1} ,\beta_{2} ,...,\beta_{p} )^{T}$ shows the vector of regression coefficients, and $p$ indicates the number of predictor variables.

### Negative Binomial (NB) regression model

In practice, the variability of the response variable is often greater than the expected value (over-dispersion), in which case the use of the Negative Binomial regression model is common [14]. If $Y_{i}$ as a random variable has the Negative Binomial distribution with the mean parameter $\mu_{i}$ and the shape parameter $k$ (to control over-dispersion), its probability density function is defined as follows:

3 $$f(Y_{i} = y_{i} ;\mu_{i} ,k) = \frac{{\Gamma (y_{i} + k)}}{{\Gamma (k)\Gamma (y_{i} + 1)}}\left( {\frac{{\mu_{i} }}{{\mu_{i} + k}}} \right)^{{y_{i} }} \left( {\frac{k}{{\mu_{i} + k}}} \right)^{k} \,\,\,\,;y_{i} = 0,1,2,...$$

With mean $E(Y_{i} ) = \mu_{i} \,$ and variance $V(Y_{i} ) = \mu_{i} + \frac{{\mu_{i}^{2} }}{k}$.

The Negative Binomial regression model is defined as the Poisson regression model (Eq. (2)).

### Zero-inflated (ZI) regression models

Zero-inflated models for high-zero data were proposed by Mullahy in 1986 [15]. The zero-inflated count model is a mixed model in which one component is count and the other is zero-degenerated. The count component is usually modeled as the Poisson distribution or Negative Binomial. Lambert in 1992 proposed the zero-inflated Poisson (ZIP) model with an application to defects in manufacturing [16]. The zero-inflated Negative Binomial (ZINB) model was introduced as a generalization of the Negative Binomial model to include large zeros in the data by Mwalili et al. in 2008 [17]. The density of the ZI model is expressed as follows ($0 < \phi < 1$):

4 $$\Pr [Y = n] = \left\{ {\begin{array}{*{20}c} {\phi + (1 - \phi )\Pr [Y = 0]} & {for\,\,\,n = 0\,\,\,\,\,\,\,\,} \\ {(1 - \phi )\Pr [Y = n]} & {for\,\,\,n = 1,2,...} \\ \end{array} } \right.\,$$

where the random variable Y follows a standard distribution such as Poisson or Negative Binomial and $\phi$ represents the uncertainty parameter (mixing proportion).

### Penalized methods

Various penalty methods such as LASSO, SCAD, ALASSO, and MCP have been proposed for variable selection. In penalty methods, removing the predictor variables not related to the response variable increases the interpretability of the model and reduces the over-fitting of the data. These methods penalize the regression coefficients in the likelihood function and select the variables by setting some coefficients to zero. In other words, the estimation of regression coefficients is obtained by minimizing the logarithm of the penalized likelihood function. Thus, variable selection and estimation of regression coefficients do simultaneously [18]:

5 $$\widehat\beta\,_{\mathbf{Penalized}\,\,\mathbf{method}}=\arg\;\min\nolimits_{\mathrm\beta}\left(-\underbrace{l(\beta)}_{\mathbf{Log}\,\boldsymbol-\mathbf l\mathbf i\mathbf k\mathbf e\mathbf l\mathbf i\mathbf h\mathbf o\mathbf o\mathbf d}+\sum_{j=1}^k\underbrace{P(\beta_j)}_{\mathbf{Penalty}\,\,\mathbf{Function}}\right)\,$$

where $l(\beta )$ is the logarithm of the likelihood function (the function can be considered PR, NB, ZIP, and ZINB), $P(.)$ is the penalty function, $\beta = \left( {\beta_{1} ,\beta_{2} ,...,\beta_{k} } \right)^{T}$ is the vector of regression coefficients, and $k$ is the number of explanatory variables.

### Penalty functions

**LASSO (Tibshirani (1996))** [19]**: **$P_{\lambda } (\beta_{j} ) = \sum\limits_{j = 1}^{k} {\lambda \left| {\beta_{j} } \right|}$, where ${{\varvec{\uplambda}}} \ge 0$ is the tuning parameter.

**SCAD (Fan and Li (2001))** [11]**: **$$p_{\lambda } (\beta_{j} ;a) = \left\{ {\begin{array}{*{20}c} {\lambda \left| {\beta_{j} } \right|} & {\left| {\beta_{j} } \right| \le \lambda } \\ { - \left( {\frac{{\beta_{j}^{2} - 2a\lambda \left| {\beta_{j} } \right| + \lambda^{2} }}{2(a - 1)}} \right)} & {\,\,\,\,\lambda < \left| {\beta_{j} } \right| \le a\lambda } \\ {\frac{{(a + 1)\lambda^{2} }}{2}} & {\left| {\beta_{j} } \right| > a\lambda } \\ \end{array} } \right.$$.

Where ${{\varvec{\uplambda}}} \ge 0$ and $a > 2$ are tuning parameters.

**ALASSO (Zou (2006))** [10]**: **$P_{\lambda } (\beta_{j} ) = \sum\limits_{j = 1}^{k} {\lambda w_{j} \left| {\beta_{j} } \right|}$, where ${{\varvec{\uplambda}}} \ge 0$ is the tuning parameter and ${\hat{\mathbf{w}}}_{j} = \frac{1}{{\left| {\hat{\beta }_{j} } \right|^{\gamma } }}$ is an adaptive weight vector ($\gamma > 0$; a possible value for $\hat{\beta }_{j}$ is the coefficients obtained from the ordinary least squares estimator method).

**MCP (Zhang (2010))** [12]**: **$$p_{a} (\beta_{j} ;\lambda ) = \left\{ {\begin{array}{*{20}c} {\lambda \left| {\beta_{j} } \right| - \frac{{\beta_{j}^{2} }}{2a}} & {\left| {\beta_{j} } \right| < a\lambda } \\ {\frac{{\lambda^{2} a}}{2}} & {\left| {\beta_{j} } \right| \ge a\lambda } \\ \end{array} } \right.$$.

Where ${{\varvec{\uplambda}}} \ge 0$ and $a > 1$ are tuning parameters.

In the present study, the tuning parameter $a$ for SCAD and MCP was considered equal to 3.7 [11, 20] and the tuning parameter λ was estimated by optimization.

### Random Forest variable selection

Random Forest introduced by Breiman (2001) is a popular algorithm and belongs to the family of ensemble methods used for both classification and regression problems [21]. This technique predicts an outcome by averaging the output of hundreds or more decision trees [22]. RF is also used as a variable selection approach in order to find informative variables [23]. In this paper, we used RF variable selection using the tree minimal depth methodology introduced by Ishwaran et al. (2010) [24].

### Simulation study

We carried out a simulation study by setting up six different scenarios to evaluate and compare the performance of five different variable selection methods. In the simulation study, we generated 100 data sets with sample sizes of 500 as a training set to optimize tunning parameters of ALASSO, SCAD, MCP, and RF. The tenfold cross-validation and out-of-bag (OOB) error were used to optimize the tuning parameters in penalized methods and the Random Forest, respectively. Furthermore, we generated additional data sets with sample sizes of 1000 in order to calculate evaluation criteria including the number of false-negative cases (NO. FN), number of true-positive cases (NO. TP), number of false-positive cases (NO. FP), number of true-negative cases (NO. TN), total accuracy (TA), sensitivity, and specificity. The number of covariates was set to 20, and 40, and they were generated from Multivariate Normal distribution with different values of correlations ($\rho = 0.2,\,\,0.5\,\,,0.7$). Five covariates were considered effective (informative covariates). For non-informative covariates, the regression coefficients were considered zero. The response variable was generated from the Negative Binomial distribution.

### Software

After entering the information recorded in the patients’ files into SPSS software (version 24) and grouping the variables, regression models were fitted using R software (version 4.1.1) by mpath, randomForestSRC, and MASS packages. Also, the pscl package was used to run the Vuong test. We used a significance level of 0.05 for all statistical analyzes.

## Results

### Simulation study

In this study, the evaluation criteria of five different variable selection methods were compared through a simulation study. The results of the simulation study (evaluation criteria) were provided in Table 1. According to the results, the sensitivity of all methods was similar and all five informative variables were selected by all methods in all scenarios. Also, the MCP tended to select a lower number of variables due to its concave penalty form, so its specificity was higher compared to the other methods. This was also the case for SCAD. Backward Stepwise and Random Forest methods tended to select more variables which resulted in a larger number of false positives and lower specificity. In general, among others, the NB-MCP had the best performance in terms of all criteria in different scenarios.

**Table 1:** Evaluation criteria for five variable selection methods over 100 repetitions by assuming Negative Binomial distribution for the response variable

Method	P	$\rho$	P'	No. FN	No. TP	No. FP	No. TN	TA	Sensitivity	Specificity
ALASSO	20	0.2	10.10 ± 1.10	0.00 ± 0.00	5.00 ± 0.00	5.10 ± 1.10	9.90 ± 1.10	0.74 ± 0.05	1.00 ± 0.00	0.66 ± 0.07
		0.5	8.20 ± 1.98	0.00 ± 0.00	5.00 ± 0.00	3.20 ± 1.98	11.80 ± 1.98	0.84 ± 0.09	1.00 ± 0.00	0.78 ± 0.13
		0.7	7.33 ± 1.80	0.00 ± 0.00	5.00 ± 0.00	2.33 ± 1.80	12.66 ± 1.80	0.88 ± 0.09	1.00 ± 0.00	0.84 ± 0.12
MCP	20	0.2	5.70 ± 2.21	0.00 ± 0.00	5.00 ± 0.00	0.70 ± 2.21	14.30 ± 2.21	0.96 ± 0.11	1.00 ± 0.00	0.95 ± 0.14
		0.5	5.10 ± 0.31	0.00 ± 0.00	5.00 ± 0.00	0.10 ± 0.31	14.90 ± 0.31	0.99 ± 0.01	1.00 ± 0.00	0.99 ± 0.02
		0.7	5.22 ± 0.44	0.00 ± 0.00	5.00 ± 0.00	0.22 ± 0.44	14.77 ± 0.44	0.98 ± 0.02	1.00 ± 0.00	0.98 ± 0.03
SCAD	20	0.2	5.00 ± 0.00	0.00 ± 0.00	5.00 ± 0.00	0.00 ± 0.00	15.00 ± 0.00	1.00 ± 0.00	1.00 ± 0.00	1.00 ± 0.00
		0.5	6.70 ± 3.43	0.00 ± 0.00	5.00 ± 0.00	1.70 ± 3.43	13.30 ± 3.43	0.91 ± 0.17	1.00 ± 0.00	0.88 ± 0.22
		0.7	6.77 ± 1.64	0.00 ± 0.00	5.00 ± 0.00	1.77 ± 1.64	13.22 ± 1.64	0.91 ± 0.08	1.00 ± 0.00	0.88 ± 0.10
Backward Stepwise	20	0.2	7.40 ± 1.07	0.00 ± 0.00	5.00 ± 0.00	2.40 ± 1.07	12.60 ± 1.07	0.88 ± 0.05	1.00 ± 0.00	0.84 ± 0.07
		0.5	7.80 ± 1.47	0.00 ± 0.00	5.00 ± 0.00	2.80 ± 1.47	12.20 ± 1.47	0.86 ± 0.07	1.00 ± 0.00	0.81 ± 0.09
		0.7	8.44 ± 1.81	0.00 ± 0.00	5.00 ± 0.00	3.44 ± 1.81	11.55 ± 1.81	0.82 ± 0.09	1.00 ± 0.00	0.77 ± 0.12
Random Forest	20	0.2	20.00 ± 0.00	0.00 ± 0.00	5.00 ± 0.00	15.00 ± 0.00	0.00 ± 0.00	0.25 ± 0.00	1.00 ± 0.00	0.00 ± 0.00
		0.5	19.90 ± 0.31	0.00 ± 0.00	5.00 ± 0.00	14.90 ± 0.31	0.10 ± 0.31	0.25 ± 0.01	1.00 ± 0.00	0.006 ± 0.02
		0.7	19.33 ± 1.00	0.00 ± 0.00	5.00 ± 0.00	14.33 ± 1.00	0.66 ± 1.00	0.28 ± 0.05	1.00 ± 0.00	0.04 ± 0.06
ALASSO	50	0.2	10.00 ± 5.21	0.00 ± 0.00	5.00 ± 0.00	5.00 ± 5.21	40.00 ± 5.21	0.90 ± 0.10	1.00 ± 0.00	0.88 ± 0.11
		0.5	9.40 ± 6.09	0.00 ± 0.00	5.00 ± 0.00	4.40 ± 6.09	40.60 ± 6.09	0.91 ± 0.12	1.00 ± 0.00	0.90 ± 0.13
		0.7	10.33 ± 4.16	0.00 ± 0.00	5.00 ± 0.00	5.33 ± 4.16	39.66 ± 4.16	0.89 ± 0.08	1.00 ± 0.00	0.88 ± 0.09
MCP	50	0.2	5.10 ± 0.31	0.00 ± 0.00	5.00 ± 0.00	0.10 ± 0.31	44.90 ± 0.31	0.99 ± 0.006	1.00 ± 0.00	0.99 ± 0.01
		0.5	5.20 ± 0.42	0.00 ± 0.00	5.00 ± 0.00	0.20 ± 0.42	44.80 ± 0.42	0.99 ± 0.008	1.00 ± 0.00	0.99 ± 0.01
		0.7	5.16 ± 0.57	0.00 ± 0.00	5.00 ± 0.00	0.16 ± 0.57	44.83 ± 0.57	0.99 ± 0.01	1.00 ± 0.00	0.99 ± 0.01
SCAD	50	0.2	5.18 ± 0.40	0.00 ± 0.00	5.00 ± 0.00	18.00 ± 0.40	44.81 ± 0.40	0.99 ± 0.008	1.00 ± 0.00	0.99 ± 0.01
		0.5	5.90 ± 2.23	0.00 ± 0.00	5.00 ± 0.00	0.90 ± 2.23	44.10 ± 2.23	0.98 ± 0.05	1.00 ± 0.00	0.98 ± 0.04
		0.7	7.91 ± 3.37	0.00 ± 0.00	5.00 ± 0.00	2.91 ± 3.37	42.08 ± 3.37	0.94 ± 0.06	1.00 ± 0.00	0.93 ± 0.07
Backward Stepwise	50	0.2	14.72 ± 2.93	0.00 ± 0.00	5.00 ± 0.00	9.72 ± 2.93	35.27 ± 2.93	0.80 ± 0.05	1.00 ± 0.00	0.78 ± 0.06
		0.5	18.50 ± 4.35	0.00 ± 0.00	5.00 ± 0.00	13.50 ± 4.35	31.50 ± 4.35	0.73 ± 0.08	1.00 ± 0.00	0.70 ± 0.09
		0.7	16.33 ± 3.14	0.00 ± 0.00	5.00 ± 0.00	11.33 ± 3.14	33.66 ± 3.14	0.77 ± 0.06	1.00 ± 0.00	0.74 ± 0.07
Random Forest	50	0.2	21.90 ± 10.08	0.00 ± 0.00	5.00 ± 0.00	16.90 ± 10.08	28.09 ± 10.08	0.66 ± 0.20	1.00 ± 0.00	0.62 ± 0.22
		0.5	17.50 ± 6.11	0.00 ± 0.00	5.00 ± 0.00	12.50 ± 6.11	32.50 ± 6.11	0.75 ± 0.12	1.00 ± 0.00	0.72 ± 0.13
		0.7	13.66 ± 1.55	0.00 ± 0.00	5.00 ± 0.00	8.66 ± 1.55	36.33 ± 1.55	0.82 ± 0.03	1.00 ± 0.00	0.80 ± 0.03

P = number of total variables, $\rho$ = correlation between variables, P’ = number of selected variables, *No. FN* Number of False-negative cases, *No. TP* Number of True-positive cases, *No. FP* Number of False-positive cases, *No. TN* Number of True-negative cases, *TA* Total Accuracy

### Schizophrenia data

**Fig. 1:**
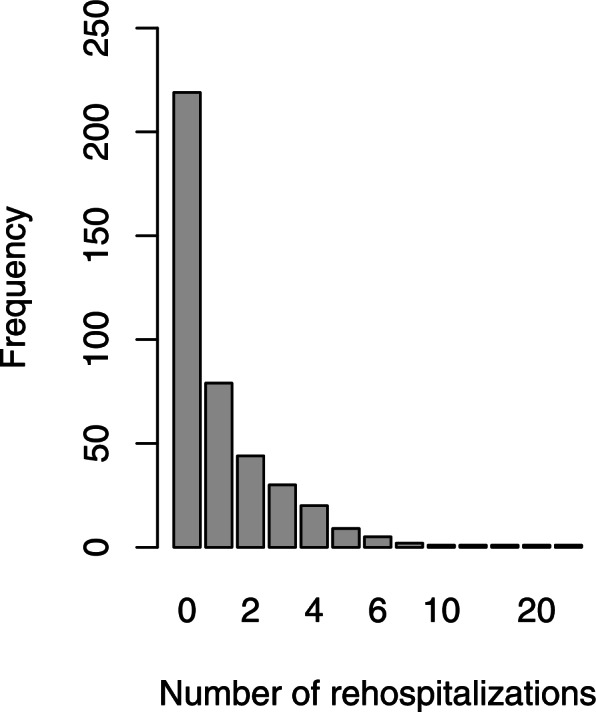
Bar plot of the number of rehospitalizations of schizophrenic patients

This study included 413 patients with schizophrenia. Table 2 shows the frequency distribution of the number of rehospitalizations. According to the results of Table 2, the number of rehospitalizations was between 0 and 21. 219 patients (53.03%) had no rehospitalization, 79 patients (19.13%) once, 44 patients (10.65%) twice, and 71 patients (17.17%) had three rehospitalizations and more. The mean (standard deviation) of the number of rehospitalizations was equal to 1.21 (2.18). The skewness coefficient of this variable was equal to 4.49 which indicates the positive skewness of its distribution. The frequency distribution of the number of rehospitalizations has appeared in Fig. 1. Table 3 shows the demographic characteristics of patients. According to Table 3, 72.60% of patients were male. The age of the patients was between 17 and 77 years with a mean (standard deviation) of 36.16 (11.18) years. Table 4 shows the clinical characteristics of the patients. According to this table, the age at onset of illness was between 5.5 and 69.5 years with a mean (standard deviation) of 26.44 (10.48) years. The duration of illness was between 0.5 and 36.5 years with a mean (standard deviation) of 9.72 (8.32) years.

**Table 2:** Frequency distribution of the number of rehospitalizations for schizophrenic patients

**Number of** rehospitalizations	**Frequency (%)**		
	**Female**	**Male**	**Sum**
0	66 (58.41)	153 (51.00)	219 (53.03)
1	28 (24.78)	51 (17.00)	79 (19.13)
2	7 (6.19)	37 (12.33)	44 (10.65)
3	5 (4.42)	25 (8.33)	30 (7.26)
4	3 (2.65)	17 (5.67)	20 (4.84)
5	2 (1.77)	7 (2.33)	9 (2.18)
6	0 (0.00)	5 (1.67)	5 (1.21)
7	1 (0.88)	1 (0.33)	2 (0.48)
10	0 (0.00)	1 (0.33)	1 (0.24)
11	0 (0.00)	1 (0.33)	1 (0.24)
13	1 (0.88)	0 (0.00)	1 (0.24)
20	0 (0.00)	1 (0.33)	1 (0.24)
21	0 (0.00)	1 (0.33)	1 (0.24)
Sum	113 (100)	300 (100)	413 (100)

**Table 3:** Description of demographic characteristics of patients with schizophrenia (*n* = 413)

**Variable**	**Variable levels**	**Frequency (%)**	**Mean ± SD**^**a**^	***P*-value**^§^
Gender	Male	300 (72.60)	0.88 ± 1.71	0.028
	Female	113 (27.40)	1.34 ± 2.33	
Age (year)	< 25	55 (13.32)	0.45 ± 0.95	< 0.001
36.16 ± 11.18	25–34	154 (37.29)	0.90 ± 1.37	
	35–44	111 (26.87)	1.68 ± 3.31	
	≥ 45	93 (22.52)	1.62 ± 1.89	
Birth season	Spring	130 (31.47)	1.11 ± 1.68	0.438
	Summer	145 (35.11)	1.43 ± 2.38	
	Autumn	67 (16.22)	0.96 ± 2.80	
	Winter	71 (17.20)	1.20 ± 1.89	
Birthplace township	Hamadan	119 (28.81)	1.02 ± 1.66	0.178
	Other Hamadan province townships	210 (50.85)	1.41 ± 2.66	
	Out of Hamadan province	84 (20.34)	1.00 ± 1.30	
Education status	Illiterate	36 (8.72)	1.42 ± 1.74	0.184
	Under diploma	237 (57.38)	1.38 ± 2.53	
	Diploma	102 (24.70)	0.85 ± 1.35	
	Academic	38 (9.20)	0.97 ± 1.93	
Marital status	Married	130 (31.48)	1.15 ± 1.59	0.924
	Separated/Divorced/Widow	50 (12.11)	1.28 ± 1.94	
	Single	233 (56.41)	1.23 ± 2.50	
Number of children	0	282 (68.28)	1.21 ± 2.39	0.023
	1–2	74 (17.92)	0.77 ± 1.24	
	≥ 3	57 (13.80)	1.82 ± 1.93	
Employment status	Employed	74 (17.92)	1.00 ± 1.92	0.165
	Housewife	45 (10.89)	0.76 ± 1.30	
	Unemployed/Disabled/Retired	294 (71.19)	1.34 ± 2.33	
Residence status	Urban	279 (67.55)	1.12 ± 2.05	0.204
	Rural	134 (32.45)	1.41 ± 2.44	
The township of residence	Hamadan	163 (39.47)	1.00 ± 1.61	0.077
	Other Hamadan province townships	197 (47.70)	1.47 ± 2.67	
	Out of Hamadan province	53 (12.83)	0.92 ± 1.49	
The population of residence (person)	< 10,000	150 (36.32)	1.42 ± 2.53	0.495
	10,000–99,000	75 (18.16)	1.07 ± 1.47	
	100,000–499,000	42 (10.17)	1.26 ± 3.23	
	≥ 500,000	146 (35.35)	1.06 ± 1.69	
Living status	With parents	229 (55.45)	1.14 ± 2.20	0.598
	With spouse	115 (27.84)	1.21 ± 1.61	
	With siblings/children/other people or alone	69 (16.71)	1.45 ± 2.86	
Having homogeneous sibling	Yes	369 (89.35)	1.25 ± 2.27	0.368
	No	44 (10.65)	0.93 ± 1.28	
Number of siblings	0–1	43 (10.41)	1.26 ± 1.54	0.197
	2–3	98 (23.73)	0.87 ± 1.44	
	≥ 4	272 (65.86)	1.33 ± 2.46	
Having a history of emotional distress	Yes	286 (69.25)	1.31 ± 2.33	0.187
	No	127 (30.75)	1.00 ± 1.79	
Having a history of arrest or prison	Yes	43 (10.41)	1.37 ± 1.67	0.615
	No	370 (89.59)	1.19 ± 2.24	
Having a history of substance abuse	Yes	145 (35.11)	1.17 ± 1.74	0.746
	No	268 (64.89)	1.24 ± 2.39	
Having a history of smoking	Yes	211 (51.09)	1.37 ± 2.29	0.125
	No	202 (48.91)	1.04 ± 2.06	

^a^ For the number of rehospitalizations

§ Independent two-sample t-test or analysis of variance test

**Table 4:** Description of clinical characteristics of patients with schizophrenia (*n* = 413)

**Variable**	**Variable levels**	**Frequency (%)**	**Mean ± SD**^**a**^	***P*-value**^§^
Age at onset of illness (year)	< 20	129 (31.24)	1.55 ± 2.64	0.134
26.44 ± 10.48	20–29	166 (40.19)	1.17 ± 2.27	
	30–39	74 (17.92)	0.88 ± 1.33	
	≥ 40	44 (10.65)	0.93 ± 1.30	
Duration of illness (year)	< 1	42 (10.17)	0.02 ± 0.15	< 0.001
9.72 ± 8.32	1–9	213 (51.57)	0.79 ± 1.23	
	10–19	92 (22.28)	1.68 ± 2.53	
	≥ 20	66 (15.98)	2.67 ± 3.51	
Having a family history of psychiatric illness	Yes	181 (43.83)	1.54 ± 2.76	0.011
	No	232 (56.17)	0.96 ± 1.55	
Having a history of medical disease	Yes	177 (42.90)	1.36 ± 2.11	0.233
	No	236 (57.10)	1.10 ± 2.24	
Having a history of non-adherence to antipsychotic drugs	Yes	303 (73.37)	1.21 ± 2.33	0.982
	No	110 (26.63)	1.21 ± 1.71	
Having a history of the suicide attempt	Yes	81 (19.61)	1.47 ± 2.57	0.240
	No	332 (80.39)	1.15 ± 2.08	

^a^ For the number of rehospitalizations

§ Independent two-sample t-test or analysis of variance test

The lowest and the highest rate of missing values with 0.72% and 6.77% belonged to the variables of history of medical disease and family history of psychiatric illness, respectively. To estimate the missing data of the variables, we used a simple imputation method (mean for imputing quantitative variables and median for imputing qualitative variables). Imputation of these missing values with a simple method is expected not to be problematic [25].

**Fig. 2:**
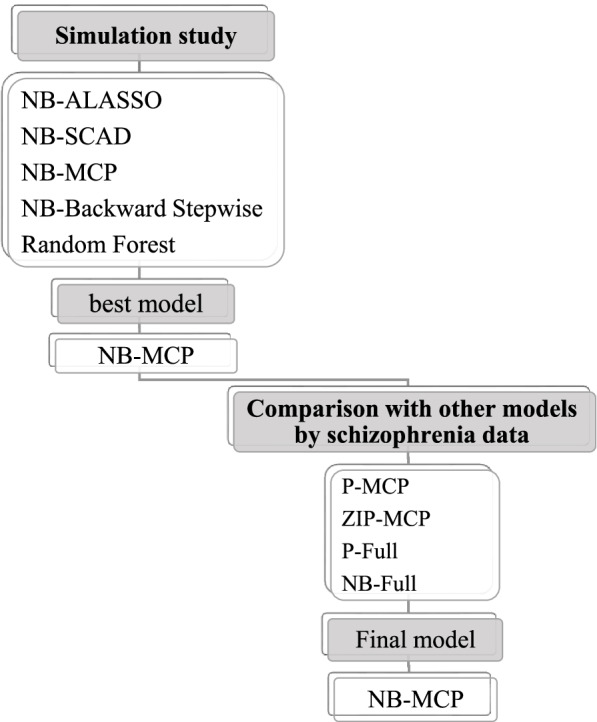
Hierarchy chart of fitted models

Table 5 shows the results of the comparisons of the NB-MCP model and other models according to the Vuong test which is a kind of likelihood ratio test [26]. It is worth noting that the fitting of the ZINB-Full, ZIP-Full, and the ZINB-MCP models did not lead to convergence. It, therefore, was not possible to compare these models with NB-MCP. According to the results of Table 5, the NB-MCP had a significantly better fit than P-MCP, P-Full, and NB-Full, however, it had a slightly better fit than ZIP-MCP. The NB-MCP model, therefore, was considered the final model. Figure 2 has summarized the fitted models through a simulation study and schizophrenia data. We also compared the performance of NB-MCP and RF based on the mean of RMSE (Root mean square error) through nested cross-validation for schizophrenia data (the Vuong test could not be applied for the RF). The Mean ± SD of RMSE for NB-MCP and RF were equal to 2.04 ± 0.41 and 1.68 ± 0.34 (*P* = 0.053), respectively. The figure of variable importance of selected variables by the Random Forest method is provided in Additional file 1 (Figure A1). Apart from that, we fitted logistic regressions with ALASSO, SCAD, MCP, Backward Stepwise, and RF with a binary response (having/ not having rehospitalization). The final logistic regression model is available in Additional file 2.

**Table 5:** Results of comparisons of NB-MCP model with other models using the Vuong test on schizophrenia data

BIC_corrected_	Vuong z-statistic	*P*-value
P-MCP	2.85	0.002^c^
ZIP-MCP	1.54	0.06
ZINB- MCP	-	-
NB-full	14.3	< 0.001^c^
P-full	6.14	< 0.001^c^
ZINB-full	-	-
ZIP-full	-	-

^c^ NB-MCP model has a significantly better fit

It should be noted that due to the strong correlation between the variables of age and age at onset of illness (*r* = 0.70), the age variable was not used in modeling the response variable. As mentioned before, the penalized variable selection methods can be used in the presence of a large number of predictor variables. Therefore, in addition to the main effects, several interactions examined in other studies were used to model the response variable. These effects were: interactions between gender and education status, gender and history of the suicide attempt, and gender and history of antipsychotic drug use. Additionally, some variables due to their clinical importance entered into the final model including the history of non-adherence to antipsychotic drugs, substance abuse, and smoking.

#### Fitting the final model

We fitted a Negative Binomial regression model (without penalty) with variables selected by NB-MCP. Table 6 shows the regression coefficients of this model. According to the results of the table, duration of illness, family history of psychiatric illness, number of children, employment status, the township of residence, and history of arrest or prison were significantly related to the number of rehospitalizations. So that longer duration of illness increased the average number of rehospitalizations $e^{0.08} = 1.08$ times. Having a positive family history of psychiatric illness increased the average number of rehospitalizations $e^{0.35} = 1.41$ times. Having at least three children compared to one or two children increased the average number of rehospitalizations $e^{0.65} = 1.91$ times. Unemployment, disability, and retirement compared to having a job increased the average number of rehospitalizations $e^{0.47} = 1.59$ times. Residence in other Hamadan province townships compared to the residence in Hamadan township increased the average number of rehospitalizations $e^{0.45} = 1.56$ times and having a positive history of arrest or prison increased the average number of rehospitalizations $e^{0.54} = 1.71$ times.

**Table 6:** Associated factors with the number of rehospitalizations among schizophrenic patients based on an NB model using selected variables by NB-MCP

Variable	Multivariate				Univariate			
	**Estimate**	**SE**	**Z statistic**	***P*-value**	**Estimate**	**SE**	**Z statistic**	***P*-value**
**Intercept**	-1.34	0.36	-3.66	< 0.001				
**Gender**								
Female (Reference category)								
Male	0.43	0.23	1.81	0.07	0.42	0.18	2.27	0.022
**Duration of illness (year)**	0.08	0.008	9.66	< 0.001	0.08	0.008	9.76	< 0.001
**Having a family history of psychiatric illness**								
No (Reference category)								
Yes	0.35	0.15	2.38	0.017	0.47	0.16	2.97	0.002
**Having a history of non-adherence to antipsychotic drugs**								
No (Reference category)								
Yes	-0.08	0.16	-0.53	0.592	0.004	0.18	0.02	0.98
**Number of children**								
3 < = (Reference category)								
0	-0.17	0.21	-0.8	0.42	-0.41	0.22	-1.85	0.064
1–2	-0.65	0.26	-2.47	0.013	-0.86	0.28	-3	0.002
**Employment status**								
Unemployed/Disabled/Retired (Reference category)								
Employed	-0.47	0.21	-2.23	0.025	-0.29	0.21	-1.34	0.18
Housewife	-0.07	0.33	-0.23	0.813	-0.57	0.28	-2.03	0.041
**The township of residence**								
Hamadan (Reference category)								
Other Hamadan province townships	0.45	0.15	2.88	0.003	0.38	0.17	2.21	0.026
Out of Hamadan province	-0.05	0.25	-0.21	0.83	-0.07	0.26	-0.29	0.769
**Having a history of arrest or prison**								
No (Reference category)								
Yes	0.54	0.24	2.28	0.022	0.13	0.26	0.53	0.594
**Having a history of substance abuse**								
No (Reference category)								
Yes	-0.12	0.21	-0.58	0.562	-0.06	0.16	-0.35	0.719
**Having a history of smoking**								
No (Reference category)								
Yes	0.25	0.21	1.19	0.232	0.27	0.16	1.7	0.089
θ	0.99	0.15						
AIC	1151							
BIC	1211.33							
Log-likelihood	-560.49							

*SE* Standard Error, *θ* Dispersion parameter

## Discussion

Identifying factors associated with the number of rehospitalizations of schizophrenia and the frequency of rehospitalizations are vitally important. In this study, several variable selection methods were used. Negative Binomial regression with MCP penalty showed a better fit.

The findings of this study showed that gender was not significantly associated with the number of rehospitalizations. However, in the study by Avcı, the male gender was significantly associated with a greater number of hospitalization [27]. Apart from that, in the studies of Kal et al. [28] and Hui et al. [29], the female gender was considered as a risk factor for relapse. This is because men and women with schizophrenia may be different in terms of age at onset, premorbid functioning, symptoms level, and outcomes [30].

In our study, a longer duration of illness was significantly associated with an increase in the number of rehospitalizations. This finding was also seen in the study of San et al. [31]. However, the study conducted by Kal et al. was inconsistent with our study [28]. Studies have shown that prolonged duration of untreated illness is significantly associated with more severe positive and negative symptoms and poorer social functioning in patients [32, 33]. The prolonged duration of untreated illness is one of the main reasons for the increase in the duration of illness. Evidence has shown that long duration of untreated illness and duration of illness are associated with poor response to therapy, especially in schizophrenia [34]. Therefore, due to this issue, it is expected that timely diagnosis and treatment of schizophrenic patients will play a crucial role in responding to their treatment and recovery.

In this study, a significant relationship was found between a positive family history of psychiatric illness and an increase in the risk of rehospitalization. According to previous studies, schizophrenic patients with this history had more severe symptoms and a higher risk of relapse and readmission [35, 36]. Esterberg et al. conducted a systematic review study and found that schizophrenic patients with a positive family history of psychosis had a significantly lower age at onset. However, in some studies, family history did not have a significant effect on the age at onset of illness, and even an inverse relationship was observed between these two variables. Having this history can also act as an environmental stressor through a conflict between the affected family member and the patient [37]. The study by Goldberg et al. was another study that found a direct link between a positive family history of psychiatric illness and the early onset of schizophrenia [38]. In the present study, the mean age of onset was significantly lower in patients with a positive family history of psychiatric illness.

Non-adherence to treatment is also one of the most important issues related to the number of rehospitalizations of schizophrenia disorder. The results of the study of Wu et al. [39] and several other studies including studies of Kal et al. [28] and Hui et al. [29] showed that drug non-compliance is significantly associated with an increased risk of relapse of this disease. Having said that in the studies of San et al. and Suzuki et al., there was no significant relationship between drug adherence and disease recurrence [31, 40] similar to our study. This can be attributed to the low percentage of drug adherence among patients (27%). On the other hand, despite the long-term use of antipsychotic drugs, there is still a possibility of recurrence of the disease [41].

Having more children was significantly associated with an increase in the number of rehospitalizations in our study. Typically, older patients also have more children. This significance therefore may be due to the relationship between the number of children and the age of patients. In this study, age groups under 25, 25 to 34, 35 to 44, and 45 years and older have an average (standard deviation) of the number of children equal to 0.05 (0.29), 0.24 (0.65), 0.81 (1.14) and 2.46 (2.21) respectively. On the other hand, having more children imposes more economic and psychological burdens on patients and their families. Supplying economic security for the family is generally the responsibility of men. In the present study, the majority of patients were male, and because patients with this disorder often face occupational and housing problems [41], they often fail to play their social roles, which can lead to feelings of shame and conflict between patients and their families [42]. Studies have shown that criticism and emotional conflicts in the family environment lead to a poor prognosis in these patients [43].

Another result of this study was that employed people had significantly fewer rehospitalizations than unemployed, disabled, and retired patients. Several studies are showing that unemployment and poor job performance are associated with an increase in the risk of disease relapse of schizophrenia disorder [39, 44, 45]. Scientific evidence shows that almost all people with schizophrenia have significant disabilities in various dimensions of function [41, 46]. Lack of attention to improving patients’ performance results in problems related to work, housing, etc. [41]. Because the early onset of this disease disrupts a person’s social and cognitive development and leads to poor social and occupational performance [47], necessary measures ought to be taken in the early years of the disease to rehabilitate patients.

According to the results of this study, the number of rehospitalizations of the disease in patients living in other Hamadan province townships was significantly higher than in Hamadan township. Lack of access to specialized medical services and long distance from the center of the province to get these services results in patients referring to the center of the province after the aggravation of the disease symptoms when there is no possibility of recovery on an outpatient basis and as a result, it increases the number of psychiatric hospitalizations. Studies have shown that timely follow-up after the discharge of schizophrenic patients can reduce the risk of readmission [48, 49]. Environmental factors such as the existence of a supportive environment and access to mental health services are considered as facilitators in the recovery of the patient, while the stigma of mental illness and interrupted health services are considered as obstacles to the patient’s recovery [50]. It thus is necessary to make appropriate plans for the evaluation and outpatient examination of patients after discharge from the hospital.

The other result of the present study was that patients who had a positive history of arrest or prison experienced a significantly higher rehospitalization rate than those who had not. In general, substance abuse is considered one of the causes of violent behavior among individuals with mental illness, and this increases the probability of being arrested and imprisoned [51]. These patients do not receive adequate medical care during their incarceration, and when they release from prison, face problems such as unemployment, homelessness, and limited access to medical care, and ultimately suffer from adverse clinical outcomes [52]. Apart from that, substance abuse exacerbates patients’ problems and leads to the relapse of disease and recurrence of the crime [53]. In our study, 81.40% of patients with a positive history of arrest or prison also had a positive history of substance abuse, however, there was no significant association between the history of substance abuse and the number of rehospitalizations.

The last result of the current study was that there was no significant association between the history of smoking and an increase in the number of rehospitalizations. However, in the study conducted by Tang et al. smokers with schizophrenia compared to patients who were non-smokers had more probability to have an episodic and more severe course and also had a significantly higher number of psychotic relapses [54]. In addition, Apud et al. found that treatment-resistant schizophrenic patients who were smokers were more apparently functionally impaired and had more positive symptoms in comparison to non-smokers [55].

The performance of statistical methods used in this study has been investigated by several studies in terms of selecting important variables. Xie and Xiao (2020), by simulating on german health care demand data, confirmed the superiority of the penalized Negative Binomial regression model compared to the full model in terms of stability in selecting predictor variables and providing a sparse model [56]. Malick and Tiwari (2016) utilized six methods including PR, NB, ZIP, and ZINB models, the LASSO method of Wang et al. [57], and the ALASSO method with a new approach on genetic data. According to their results, the ZINB model with the new approach of the ALASSO method provided the best fit for identifying SNPs related to the number of phenotypes [58]. Wang et al. (2015) used the ZINB model with three penalties including LASSO, SCAD, and MCP to model the number of doctor office visits. The ZINB-MCP model was selected as the best-penalized model and had a better prediction compared to the NB-MCP, ZINB-Full, and ZINB-Backward Stepwise models [57]. Wang et al. (2014) used the ZIP regression and three penalties including LASSO, SCAD, and MCP to identify the factors affecting the number of postoperative morbidity after cardiac surgery and the ICU length of stay. They found that the ZIP-MCP model fit was better than the ZIP-Backward Stepwise model in analyzing the length of stay of patients in the ICU. In addition, ZIP-MCP and ZIP-SCAD models did not show a significant difference from the ZIP-Full model. Analysis of the number of postoperative morbidity after cardiac surgery also led to similar results, with the difference that the software failed to fit the ZIP-Full model and could not fit the model with all predictor variables in the two components of zero and Poisson [59]. In the present study, the fit of the ZINB-Full and ZIP-Full models did not lead to convergence. It thus was not possible to fit ZINB-Backward Stepwise and ZIP-Backward Stepwise models. Consequently, when the zero-inflated full regression model faces the problem of non-convergence [57, 60] and we are trying to find a sparse model, it is necessary to use penalized variable selection methods or other variable selection methods such as Random Forest instead of classical methods such as the stepwise. Zeng et al. (2014) utilized the ZIP & ZINB regression models and ALASSO method to identify the factors affecting the number of doctor visits. The simulation results showed the identification of important variables by applying this penalty method [61]. Buu et al. (2011) compared the PR-LASSO, PR-SCAD, ZIP-LASSO, and ZIP-SCAD models in terms of the identification of risk factors for substance abuse and alcoholism. Finally, the ZIP-SCAD model showed the best fit based on criteria such as sensitivity and specificity [62].

There were some limitations to the present study. For instance, the high percentage of male participants, no electronic patient records, not recorded some important variables including the father’s age at birth, the quality of family relationships, socio-economic status, etc. (that may in addition to the effect on the incidence of schizophrenia play a role in rehospitalizations of schizophrenic patients), the existence of missing values for some variables, underestimating the response variable due to the fact that some patients had not been referred to Farshchian Hospital during the study period (March 2011-March 2019) for reasons such as change of residence and not being convinced for psychiatric hospitalization, the existence of Berkson’s bias (people who have been hospitalized more are often more likely to be readmitted), the lack of resources that are similar and comparable to our study in terms of the nature of response variable and disease, non-convergence of ZIP-Full and ZINB-Full models as well as the impossibility of fitting these models with the Backward Stepwise method, non-convergence of the ZINB-MCP model, and as a result, the impossibility of comparing these non-convergent models with the best model found by simulation study (i.e., NB-MCP). Despite these limitations, given that so far almost all studies on schizophrenia have examined the occurrence or non-occurrence of the rehospitalization of this disease through logistic regression, the use of count models to identify the decreasing and increasing factors of rehospitalization can be one of the strengths of this study. Modeling the number of rehospitalizations of schizophrenia disorder through penalty methods and Random Forest was used for the first time in the present study. Models used in this research can be useful in modeling many count outcomes especially when zero-inflated full models face the problem of non-convergence and as a result, in practice, we cannot fit the classical variable selection methods. When we have a large number of predictor variables and a small sample size, the use of penalty methods provides remarkably more stable results than classical methods for variable selection. A wide range of predictor variables therefore can be examined through penalty methods. For example, in future studies, in addition to demographic and clinical factors, genetic variables can be used to model the number of rehospitalization for schizophrenia disorder. In this study, we compared the performance of penalized methods to Random Forest in terms of selecting important covariates assuming Negative Binomial distribution for the response variable. It is suggested to compare the methods used in this study with other machine learning methods such as the Support Vector Machine (SVM) and also to consider zero-inflated distribution for the response variable.

## Conclusions

The results obtained from the use of variable selection methods in this study showed that the NB-MCP model is the best model for explaining the number of rehospitalizations of schizophrenia disorder. To reduce the number of rehospitalizations in patients with schizophrenia disorder, it is recommended to provide special medical services for patients who do not have access to specialized medical centers and to create the necessary infrastructure for the employment of patients.

## Supplementary Information

**Additional file 1:Figure A1.** Variable importance of selected variables by Random Forest method.

**Additional file 2:Table A1.**Associated factors with rehospitalization among schizophrenic patients based on a Logistic model using selected variables by MCP.

## Data Availability

The data are available upon reasonable requests from the corresponding author.

## Abbreviations

*PR*: Poisson Regression
*NB*: Negative Binomial
*ZI*: Zero-inflated
*ZIP*: Zero-inflated Poisson
*ZINB*: Zero-inflated Negative Binomial
*LASSO*: Least Absolute Shrinkage and Selection Operator
*ALASSO*: Adaptive Least Absolute Shrinkage and Selection Operator
*SCAD*: Smoothly Clipped Absolute Deviation
*MCP*: Minimum Concave Penalty
*RF*: Random Forest
*AIC*: Akaike Information Criterion
*BIC*: Bayesian Information Criterion
