# Supplementary material for: Investigating factors associated with the number of rehospitalizations among patients with schizophrenia disorder using penalized count regression models
Source: BMC Med Res Methodol. 2022 Jun 15;22:170. doi: 10.1186/s12874-022-01648-z (PMC9202127; doi:10.1186/s12874-022-01648-z)
Supplement: Supplementary file 1 — Additional file 1:Figure A1. Variable importance of selected variables by Random Forest method. [file 12874_2022_1648_MOESM1_ESM.docx]

Figure A1: Variable importance of selected variables by Random Forest method

Figure A1 shows the variable importance of selected variables by Random Forest method. According to the variable importance plot, the variables of the duration of illness, age at the onset of the illness, having a history of arrest/prison, and having a history of the suicide attempt were selected as the most important variables associated with the number of re-hospitalizations among the patients.
