# Supplementary material for: Investigating factors associated with the number of rehospitalizations among patients with schizophrenia disorder using penalized count regression models
Source: BMC Med Res Methodol. 2022 Jun 15;22:170. doi: 10.1186/s12874-022-01648-z (PMC9202127; doi:10.1186/s12874-022-01648-z)
Supplement: Supplementary file 2 — Additional file 2:Table A1.Associated factors with rehospitalization among schizophrenic patients based on a Logistic model using selected variables by MCP. [file 12874_2022_1648_MOESM2_ESM.docx]

According to the results of Table A1, longer ‎duration of illness (P<0.001) was significantly associated with rehospitalization.

Table A1: Associated factors with rehospitalization among schizophrenic patients based on a Logistic model using selected variables by MCP

| **Univariate** | | | | **Multivariate** | | | | **Variable** |
| --- | --- | --- | --- | --- | --- | --- | --- | --- |
| **P-value** | **Z statistic** | **SE** | **Estimate** | **P-value** | **Z statistic** | **SE** | **Estimate** |  |
|  |  |  |  | <0.001 | -4.16 | 0.27 | -1.13 | **Intercept** |
| <0.001 | 7.26 | 0.01 | 0.11 | <0.001 | 7.16 | 0.01 | 0.11 | **Duration of illness (year)** |
|  |  |  |  |  |  |  |  | **Having a history of non-adherence to antipsychotic drugs** |
|  |  |  |  |  |  |  |  | No (Reference category) |
| 0.235 | -1.18 | 0.22 | -0.26 | 0.361 | -0.91 | 0.24 | -0.22 | Yes |
|  |  |  |  |  |  |  |  | **Having a history of substance abuse** |
|  |  |  |  |  |  |  |  | No (Reference category) |
| 0.521 | -0.64 | 0.20 | -0.13 | 0.535 | -0.62 | 0.32 | -0.20 | Yes |
|  |  |  |  |  |  |  |  | **Having a history of smoking** |
|  |  |  |  |  |  |  |  | No (Reference category) |
| 0.443 | 0.76 | 0.19 | 0.15 | 0.224 | 1.21 | 0.31 | 0.38 | Yes |

SE: Standard Error
